# Du Huo Ji Sheng Tang inhibits Notch1 signaling and subsequent NLRP3 activation to alleviate cartilage degradation in KOA mice

**DOI:** 10.1186/s13020-023-00784-y

**Published:** 2023-06-29

**Authors:** Wen-jin Chen, Yin Zhuang, Wei Peng, Wei Cui, Shu-jun Zhang, Jian-wei Wang

**Affiliations:** 1 Department of Orthopaedics, Wuxi 9th People’s Hospital Affiliated to Soochow University, No. 999 of Liangxi Road, Wuxi, 214062 China; 2 Department of Orthopaedics, Wuxi Hospital of Traditional Chinese Medicine, No. 8 West of Zhongnan Road, Wuxi, 214071 China

**Keywords:** Osteoarthritis, DHJST, NLRP3, Notch1, Mice, Inflammation

## Abstract

**Background:**

Knee osteoarthritis (KOA) has a complex pathological mechanism and is difficult to cure. The traditional medicine Du Huo Ji Sheng Tang (DHJST) has been used for the treatment of KOA for more than one thousand years, but its mechanism for treating KOA has not been revealed. In our previous study, we confirmed that DHJST inhibited the activation of NLRP3 signaling in rats and humans. In the current study, we aimed to determine how DHJST inhibits NLRP3 to alleviate knee cartilage damage.

**Methods:**

Mice were injected with NLRP3 shRNA or Notch1-overexpressing adenovirus into the tail vein to construct systemic NLRP3 low-expressing or Notch1 high-expressing mice. Mice were injected with papain into the knee joint to replicate the KOA model. DHJST was used to treat KOA model mice with different backgrounds. The thickness of the right paw was measured to evaluate toe swelling. The pathohistological changes and the levels of IL-1β, MMP2, NLRP3, Notch1, collagen 2, collagen 4, HES1, HEY1, and Caspase3 were detected by HE staining, ELISA, immunohistochemical staining, western blotting, or real-time qPCR.

**Results:**

DHJST reduced tissue swelling and serum and knee cartilage IL-1β levels, inhibited cartilage MMP2 expression, increased collagen 2 and collagen 4 levels, decreased Notch1 and NLRP3 positive expression rates in cartilage, and decreased HES1 and HEY1 mRNA levels in KOA model mice. In addition, NLRP3 interference decreased cartilage MMP2 expression and increased collagen 2 and collagen 4 levels without affecting the expression levels of notch1, HES1 and HEY1 mRNA levels in the synovium of KOA mice. In KOA mice with NLRP interference, DHJST further reduced tissue swelling and knee cartilage damage in mice. Finally, Notch1-overexpressing mice not only showed more severe tissue swelling and knee cartilage degradation but also abolished the therapeutic effect of DHJST on KOA mice. Importantly, the inhibitory effects of DHJST on the mRNA expression of NLRP3, Caspase3 and IL-1β in the knee joint of KOA mice were completely limited after Notch1 overexpression.

**Conclusion:**

DHJST significantly reduced inflammation and cartilage degradation in KOA mice by inhibiting Ntoch1 signaling and its subsequent NLRP3 activation in the knee joint.

## Introduction

Osteoarthritis (OA) is the most common chronic joint disease. Clinically, the knee joint is the most common site of osteoarthritis, with knee osteoarthritis (KOA) accounting for 85% of the global burden of osteoarthritis [1]. The pathogenesis of OA involves the destruction of articular cartilage, subchondral osteosclerosis and the formation of osteoarthritis [2]. As chondrocytes are the only cellular component of articular cartilage, chondrocyte damage is a key factor in the progression of OA[3]. Inflammation is one of the core factors leading to articular cartilage destruction and symptoms of osteoarthritis. Inflammatory cytokines such as interleukin-1β (IL-1β) induce the production of catabolic factors, such as matrix metalloproteinases (MMPs), and inhibit the synthesis of anabolic factors, such as collagen, which promotes chondrocyte death [4].

The incidence of KOA increases with age, and although the pathogenesis of KOA has not been elucidated, it can be prevented and delayed by interventions and health behaviors, especially traditional medications that have a complementary role in the treatment of KOA [5, 6]. Controlling inflammation has become an effective strategy for the treatment of KOA [7]. In our previous study, we demonstrated that the traditional formula Du Huo Ji Sheng Tang (DHJST) has an inhibitory effect on NLRP3 signaling in a rat KOA model [8]. Studies have shown that the NLRP3 inflammasome is involved in the pathogenesis of OA and can promote cartilage degeneration and synovial inflammation through functional interaction with multiple other inflammatory signaling pathways, such as Toll-like receptors and NF-κB [9, 10]. DHJST was created by Sun Simiao, a famous physician in the Tang Dynasty of China, and contains 15 herbs, including *Duhuo* and *Mulberry Jisheng*. This formula is a well-known formula for the clinical treatment of wind-cold and damp paralysis and has been used in the clinical treatment of arthritis in China for over a thousand years [11–13]. At present, there are few basic studies on DHJST, and the potential mechanism by which NLRP3 regulates to alleviate KOA is still unknown.

To further clarify the mechanism of DHJST and provide evidence for the complementary medicine clinical application of DHJST in KOA treatment. We hypothesized that DHJST could reduce cartilage degradation in inflammatory states and delay the progression of OA by inhibiting Notch1 signaling-mediated NLRP3 activation in the knee joint. Recent studies have shown that Notch1 mediates the activation of NLRP3 [14, 15]. In this study, mice were treated with NLRP3 shRNA or Notch1-overexpressing adenovirus, a mouse papain knee injection was used to induce an animal model of KOA in mice, and DHJST was used to treat the mice after modeling. We evaluated KOA development and examined alterations in the Notch1 and NLRP3 signaling pathways in mice. In the current study, we aimed to confirm the regulatory effects of DHJST on chondrocyte degradation and the NLRP3 signaling pathway and to determine whether DHJST alleviated inflammation and protected cartilage via the notch1 pathway.

## Materials and methods

### Reagents

DHJST was provided by the Preparation Department of Wuxi Hospital of Traditional Chinese Medicine. The mouse IL-1β kit (#EK201B), IL-6 kit (#EK206), and IL-18 kit (#EK218) were purchased from Hangzhou Lianke Biotechnology Joint Stock Company (Hangzhou, China). EDTA decalcification solution (#EK201B), hematoxylin eosin (HE) staining kit (#G1120) and DAB chromogenic kit (#DA1010) were purchased from Beijing Solebo Technology Co., Ltd. (Beijing, China). IL-1 beta polyclonal antibody (#16806-1-AP), MMP-2 polyclonal antibody (#10373-2-AP), collagen type II polyclonal antibody (#28459-1-AP), collagen type VI polyclonal antibody (#17023-1-AP), NOTCH1 polyclonal antibody (#20687-1-AP), NLRP3 polyclonal antibody (#19771-1-AP), Caspase-1 polyclonal antibody (#22915-1-AP), ASC polyclonal antibody (#11529-1-AP), and GAPDH monoclonal antibody (#6404-1-Ig) were purchased from Wuhan Sanying Biotechnology Co., Ltd. (Wuhan, China). The AdvanceFast 1st Strand cDNA Synthesis Kit (#11149ES60) and PCR Reaction Kit (miRNA UniversalqPCR SYBR Master Mix, #11171ES08) were purchased from Yisheng Biotechnology (Shanghai) Joint Stock Company (Shanghai, China).

### Preparation and composition analysis of DHJST

The herbs of DHJST were weighed according to the prescribed proportions from Chinese Pharmacopoeia, which consisted of 15 herbal components including Radix Angelicae Pubescentis, Herba Taxilli, Radix Eucommiae, Radix Achyranthis Bidentatae, Radix Gentianae Macrophyllae, Poria, Ramulus Cinnamomi, Radix Saposhnikoviae, Radix Angelicae Sinensis, Radix Glycyrrhizae, Radix Paeoniae Alba, Radix Paeoniae Rubra, Radix Rehmanniae Preparata, Radix Paeoniae lactiflorae, and Radix Paeoniae Rubra. The usual dose for human was prepared as a decoction with 9 g of Radix Angelicae Pubescentis and 6 g of other herbal components boiled together.

**Fig. 1:**
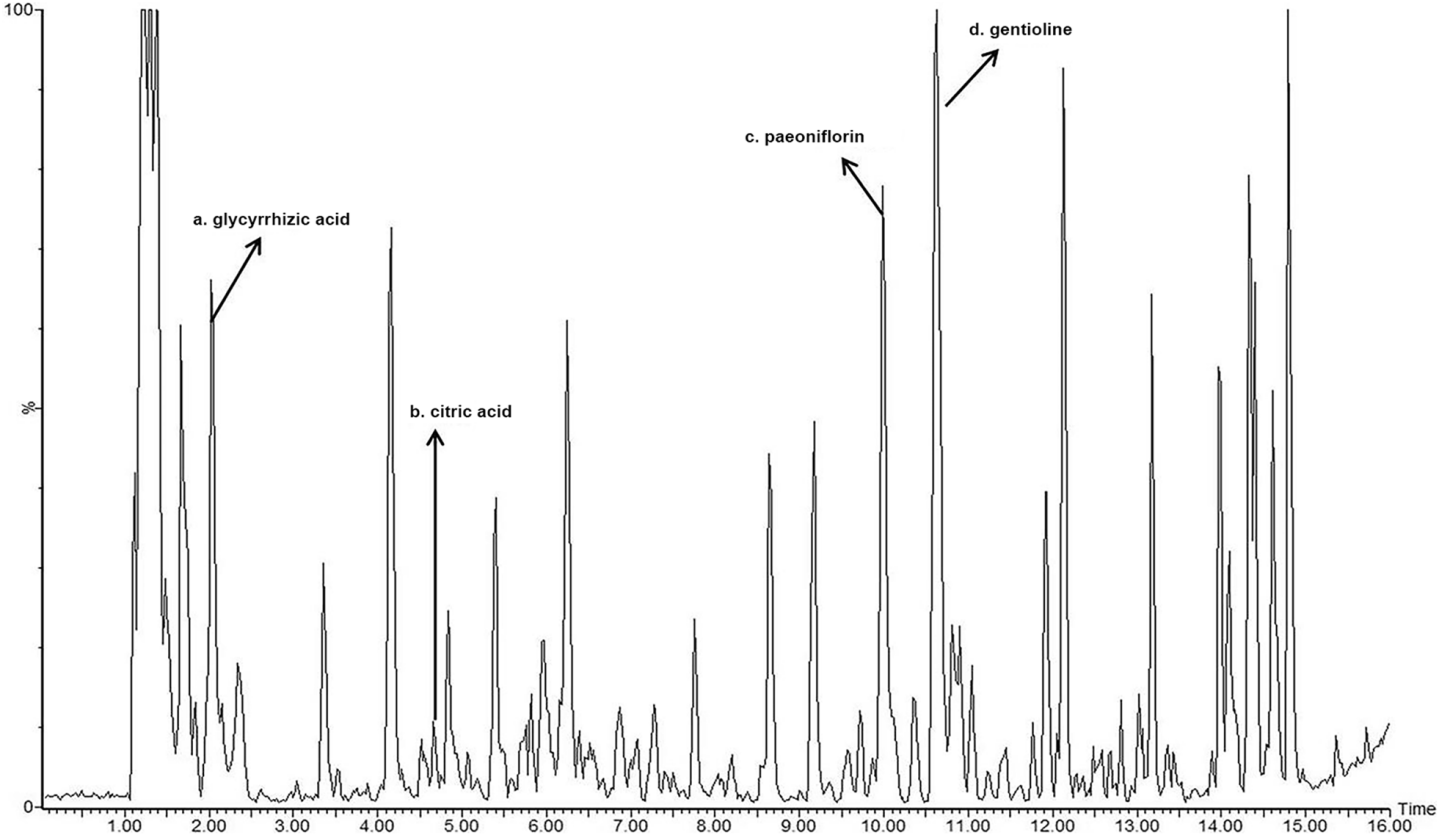
Chemical composition analysis of DHJST by UPLC method Four main components of DHJST were determined, including **a**: glycyrrhizic acid, **b**: citric acid, **c**: paeoniflorin, and **d**: gentioline.

The herbs were mixed with distilled water and soaked overnight. The material was boiled for 1 h and filtered to obtain an aqueous decoction. The aqueous decoction was concentrated and diluted with water according to the dose administered. The four major components of DHJST, including glycyrrhetinic acid, citric acid, paeoniflorin, and gentioline, were determined by UPLC, with a detection wave length of 230 nm. The retention time (t_R_) of glycyrrhetinic acid, citric acid, paeoniflorin, and gentioline were 2.12, 4.65, 10.35, 10.64 min, respectively (Fig. 1).

### Construction of adenovirus and in vivo infection in mice

Hanheng Biotechnology (Shanghai) Co., Ltd. synthesized specific shRNA targeting the mouse NLRP3 gene and mouse Notch1 overexpression plasmid and carried out adenovirus packaging and purification of interfering and overexpressing genes. According to the instructions, these adenoviruses can cause low or high expression of specific genes after being injected into mice by tail vein for approximately three to four weeks. We injected control AAV2 (Con AAV), NLRP3 shRNA AAV2 (nlr_KD AAV) and Notch1 overexpression AAV2 (notch1_OE AAV) into the tail vein of C57BL/6 mice and detected the mRNA expression levels of NLRP3 and Notch1 in the tail tip of the mice three weeks after injection. Mice with low expression of NLRP3 (more than 50%) or high expression of Notch1 (more than 1-fold) were successfully infected and were used in subsequent experiments.

### Replication and intervention of the mouse KOA model

The KOA model was constructed by injecting papain into the right knee joint cavity of mice. A 4% papain solution was prepared in physiological saline and injected once every 7 days. After 3 consecutive injections, the model was regarded as successful. After that, the mice in each group received intragastric treatment. The mice in the control group and the model group received intragastric administration of an equal volume of normal saline, while the mice in the DHJST group were given intragastric administration of the corresponding concentration of drugs once a day for 4 weeks. If the mice used were adenovirus-infected mice, the model was replicated after successful infection.

DHJST consists of 15 herbal components, including Radix Angelicae Pubescentis, Herba Taxilli, Radix Eucommiae, Radix Achyranthis Bidentatae, Radix Gentianae Macrophyllae, Poria, Ramulus Cinnamomi, Radix Saposhnikoviae, Radix Angelicae Sinensis, Radix Glycyrrhizae, Radix Paeoniae Alba, Radix Paeoniae Rubra, Radix Rehmanniae Preparata, Radix Paeoniae lactiflorae, and Radix Paeoniae Rubra. The usual dose for human is prepared as a decoction with 9 g of Radix Angelicae Pubescentis and 6 g of other herbal components boiled together. The decoction can be taken for 1–3 days depending on the severity of symptoms, and the mid-value of 2 days is used as the human dosage, which is 3 g of Radix Angelicae Pubescentis and 2 g of other herbal components per day, with a total weight of 31 g. To convert the dosage for mice, a body surface area conversion factor of 0.0026 for human and mouse and a mouse weight of 20 g were used. Therefore, the high dosage for mice was defined as 4 g/kg and the low dosage as 2 g/kg of DHJST.

### Grouping of mice and joint swelling detection

A total of three independent animal groupings were used in this study. The first group was the control group, model group, DHJST low-dose (2 g/kg) group and DHJST high-dose (4 g/kg) group. The second group included the Con AAV model group, Con AAV DHJST (4 g/kg) group, nlr_KD AAV model group, and nlr_KD AAV DHJST (4 g/kg) group. The third group was the Con AAV model group, Con AAV DHJST (4 g/kg) group, notch1_OE AAV model group, and notch1_OE AAV DHJST (4 g/kg) group. In the three independent experiments, the number of animals in each group was 8.

Before the injection of papain, the thickness of the center point of the mouse’s right paw was measured with a Vernier caliper. After 3 injections of papain, the thickness of the center point of the right paw of mice was measured again using a Vernier caliper. After starting the treatment, the thickness of the center point of the mouse’s right paw was measured with a Vernier caliper every 7 days until the end of DHJST treatment. The thickness of the right paw was measured 6 times in each group of mice during the whole experimental period to evaluate the degree of joint swelling.

### ELISA of mouse serum IL-1β, IL-6, and IL-18

The mouse serum of each group was collected, referring to the assay instructions of the mouse IL-1β, IL-6, and IL-18 ELISA kits, and serum and standard substances were added. After incubation for 2 h, the color development reagent was added, and the reaction plate was washed 5 times after color development termination. A microplate reader was used to determine the absorbance value at 450 nm for each well. The standard curves were fitted, and the levels of IL-1β, IL-6, and IL-18 in the serum of each mouse were calculated.

### Knee joint bone and synovial membrane preparation and H&E staining

The tibia and synovium of the mouse were collected and fixed in paraformaldehyde. The fixed tibia was washed with water, decalcified in Ethylenediaminetetraacetic acid (EDTA) solution for 6 weeks, and then dehydrated in different concentrations of ethanol (70%, 80%, 90%, 95%, 100%, and 100%). The synovium of the mouse knee joint could be directly dehydrated after being fixed. After the mouse tibial cartilage or knee synovium was embedded in paraffin, the sections were prepared using a paraffin microtome. The tibial cartilage sections of each mouse were stained with HE staining solution after routine dewaxing and rehydration. Photographs were taken with a light microscope after sealing, and the obtained images were used for general histological evaluation. Score the severity of joint inflammation and damage according to a scoring system based on the degree of damage. Assign scores of 0–5 points based on the following criteria: 0 points: no observable damage; 1 point: mild damage, such as mild synovial hyperplasia or slight inflammatory cell infiltration; 3 points: moderate damage, such as moderate synovial hyperplasia, moderate inflammatory cell infiltration, or mild cartilage erosion; 5 points: severe damage, such as severe synovial hyperplasia, severe inflammatory cell infiltration, or extensive cartilage and bone erosion. Analyze the pathological scores and compare them between the different treatment groups to assess the severity of joint damage.

### Immunohistochemical staining of knee cartilage and synovial membrane

Tibial cartilage or knee synovium sections from mice were immersed in hydrogen peroxide solution for 10 min after routine dewaxing and rehydration. After 3 washes, sodium citrate antigen repair solution was used for heat repair of antigens in the tissues. After washing 3 times, mouse sections were placed flat in a humidor. Then, 5% BSA was incubated for 1 h to block the antigen, and after blocking, tibial cartilage tissues were incubated with diluted (1:100) IL-1 beta polyclonal antibody, MMP-2 polyclonal antibody, NOTCH1 polyclonal antibody, and NLRP3 polyclonal antibody; and knee synovial tissues were incubated with diluted (1:100) MMP-2 polyclonal antibody, NOTCH1 polyclonal antibody, NLRP3 polyclonal antibody, Caspase-1 polyclonal antibody, and ASC polyclonal antibody.

After overnight incubation at 4 °C, the mouse tissues were washed 3 times, and the tibial cartilage or knee synovium was incubated with HRP-modified goat anti-rabbit IgG (1:200) for 2 h. After washing again 3 times, the sections were stained using the DAB color development kit. Ten minutes later, the mouse tissue sections were washed 3 times and sealed. The sections were photographed using a light microscope, and the obtained images were analyzed using Image-Pro Plus software for mean optical density assessment.

### Western blot of knee cartilage

Western blotting was performed according to our previous study [8]. Cryopreserved mouse femoral cartilage tissue was lysed by electrokinetic homogenate. Then, the cell debris was removed by centrifugation at 12,000 rpm for 10 min at 4 °C. The protein concentration level was determined by the BCA kit. An equal amount of the extracted total protein (50 µg) was separated by SDS‒PAGE, and then the protein in the gel was transferred to a PVDF membrane. The PVDF membrane was preincubated at room temperature in 5% blocking buffer for 30 min and then incubated with diluted collagen type II polyclonal antibody (1:1000) and collagen type VI polyclonal antibody (1:1000) overnight at 4 °C. Subsequently, the membrane was incubated with secondary antibody (1:10000) for 2 h and then washed 3 times with TBST. The blot was quantified using a gel imaging system and an ultrasensitive ECL luminescence kit.

### Real-time qPCR of knee synovial membrane

Total RNA was extracted from mouse knee synovial tissue using TRIzol reagent. mRNA was transcribed into cDNA using a reverse transcription reagent inverse kit, and experiments were performed using a real-time fluorescent quantitative PCR mixture in a real-time fluorescent quantitative PCR system using the following primers. All of the primers were synthesized by Sangon Biotech (Shanghai) Co., Ltd. NLRP3 (forward 5′- ATTACCCGCCCGAGAAAGG-3′, reverse 5′- TCGCAGCAAAGATCCACACAG-3′) 141 bp. HES1 (forward 5′-GATAGCTCCCGCATTCCAAG-3′, reverse 5′-GCGCGGTATTTCCAACA-3′) 134 bp. HEY1 (forward 5′-GCCCTGGCTATGGACTATCG-3′, reverse 5′-CGCTGGGATGCGTAGTTGT-3′) 146 bp. Caspase3 (forward 5′-TGAAGGGGTCATTTATGGGACA − 3′, reverse 5′-CCAGTCAGACTCCGGCAGTA − 3′) 90 bp. IL-1β (forward 5′-TGATGTGCTCACTGCCTGGTTTC-3′, reverse 5′-GTTGATGTGCTGCTGCGAGATTTG-3′) 97 bp. GAPDH (forward 5′-ACCACAGTATCCATGCCATCAC-3′, reverse 5′-TCCACCACCCTGTTGCTGTA-3′) 453 bp. The GAPDH gene was used as an endogenous control to normalize the difference in total RNA amount. The 2^−ΔΔct^ method was used to calculate the relative expression levels of various inflammatory factors in mouse cartilage.

### Statistical analysis

All results were analyzed and plotted using GraphPad 9.0 and are expressed as the mean ± label difference. For multiple between-group comparisons, one-way ANOVA was used for the test of variance, and multiple comparisons between groups were performed with Tukey’s post hoc test. P < 0.05 was considered statistically significant.

## Results

### DHJST improves toe swelling, joint injury and inflammation in KOA mice

**Fig. 2:**
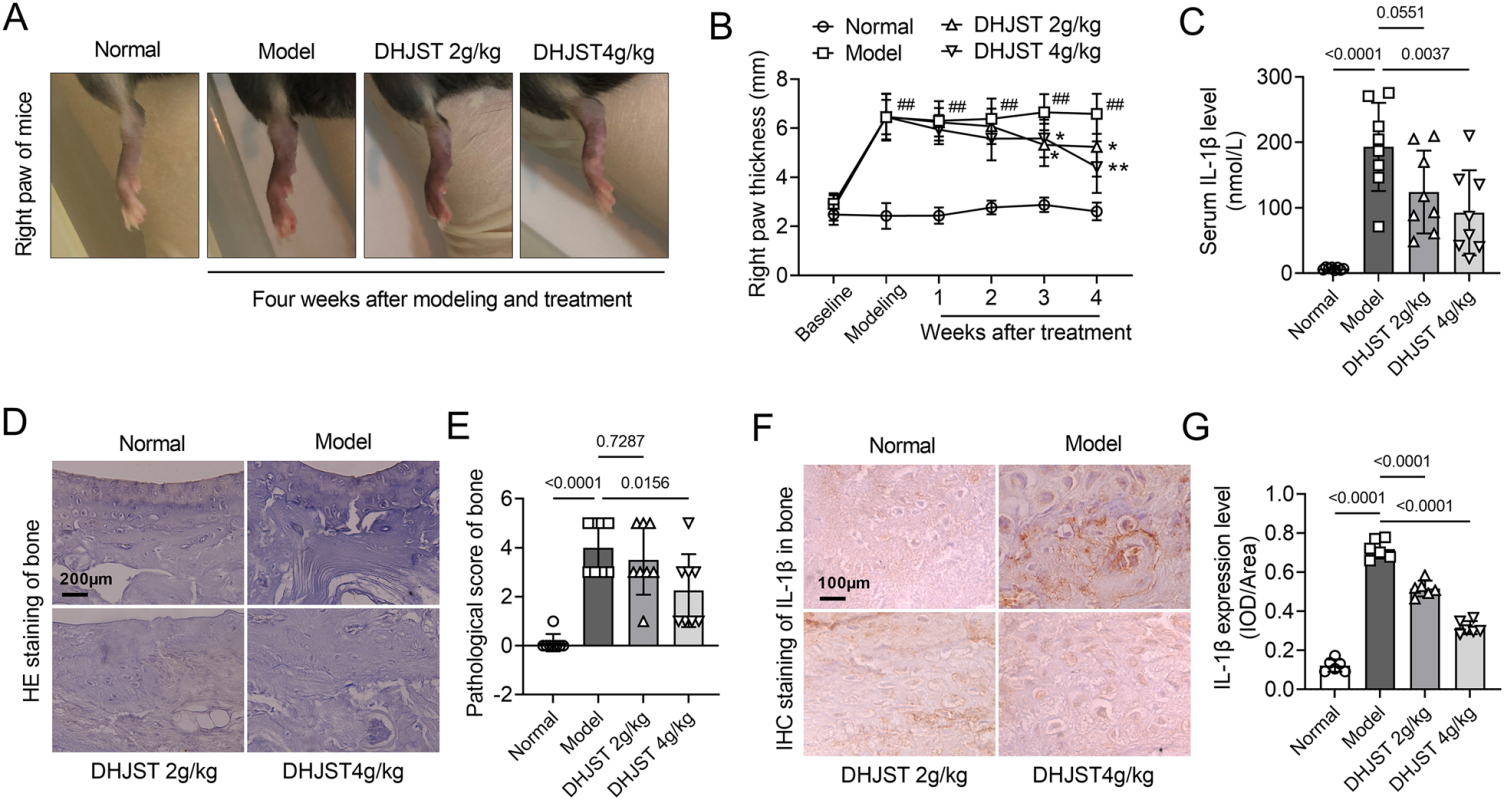
DHJST improves toe swelling, joint injury and inflammation in KOA mice C57BL/6 mice were injected with 4% papain 0.1 ml (once a week for three weeks) into the right knee joint to induce KOA. After that, they were treated with 2 g/kg or 4 g/kg DHJST for four weeks. **A** Representative pictures of the morphology of the right knee joint of the mice after treatment. **B** Line plots recorded the thickness of the right paw of the mice before modeling (baseline), after modeling (modeling), and after weekly administration (1–4 weeks). Compared with the normal group, #P < 0.05; compared with the model group, * P < 0.05. **C** Detection of serum IL-1β levels in mice by ELISA kit. **D**, **E** HE staining of the right knee joint of mice and its pathological score. **F**, **G** Immunohistochemical staining of IL-1β in the cartilage of the right knee joint of mice and its mean optical density analysis. N = 8 in **A**–**E**, N = 6 in **F**, **G**

Compared with normal mice (normal group), the right foot of the mice in the other groups was significantly swollen after 3 weeks of papain injection. Compared with the model mice (model group), the toe volume of the mice was significantly reduced after 3 weeks of treatment with DHJST. At the 4th week of treatment, the degree of toe swelling of the mice continued to decrease (P < 0.05, Fig. 2A, B). Compared with the normal group, the serum IL-1β level of the mice in the model group was significantly increased, and the serum IL-1β level was significantly reduced after 4 weeks of treatment with DHJST (4 g/kg) (Fig. 2C). HE staining showed that the chondrocytes in the normal group were horizontally arranged and the articular cartilage edge was smooth, while the articular cartilage edge in the model group was severely damaged and the chondrocyte arrangement was disordered. The cartilage structure of the mice in the DHJST treatment group tended to be normal. The pathological score of the KOA model group was significantly higher than that of the normal group (P < 0.0001). Compared with the model group, the pathological score of the mice in the DHJST (4 g/kg) group was significantly lower (P = 0.0156) (Fig. 2D, E). Immunohistochemical staining of IL-1β in the knee cartilage of mice showed that the positive expression rate of IL-1β in the cartilage of mice in the model group was significantly higher than that in the normal group (P < 0.0001). Treatment with DHJST (2 g/kg or 4 g/kg) reduced the positive expression rate of IL-1β in the cartilage of KOA model mice (P < 0.0001, P < 0.0001) (Fig. 2F, G).

### DHJST reduces cartilage degradation in KOA mice

**Fig. 3:**
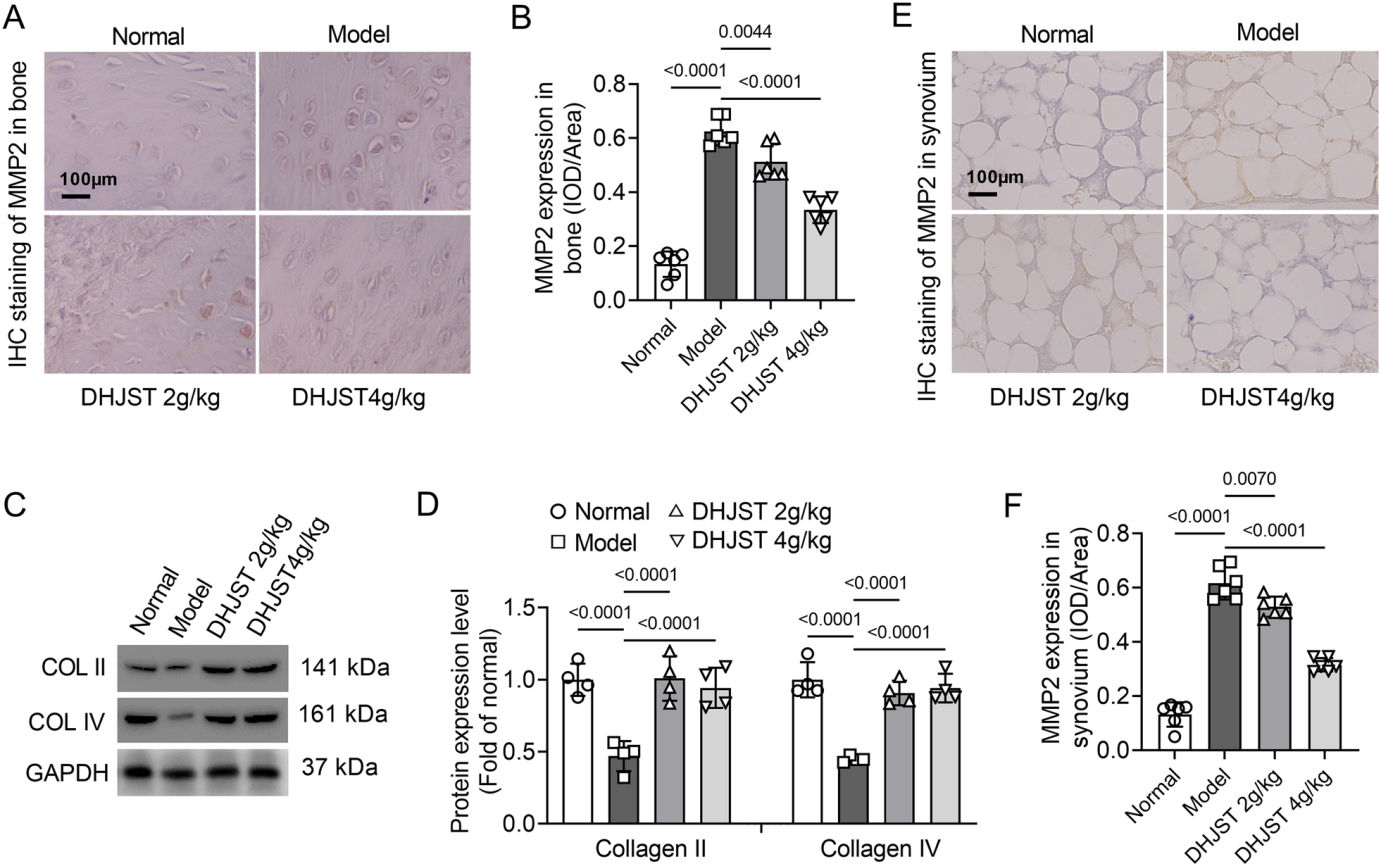
DHJST reduces cartilage degradation in KOA mice C57BL/6 mice were injected with 4% papain 0.1 ml (once a week for three weeks) into the right knee joint to induce KOA. After that, they were treated with 2 g/kg or 4 g/kg DHJST for 4 weeks. **A**, **B** Immunohistochemical staining of MMP2 in the right knee cartilage of mice and its mean optical density analysis, N = 6. **C**, **D** Western blot detection of collagen 2 and collagen 4 in the right knee cartilage of mice and the analysis of their relative expression levels, N = 4. **E**, **F** Immunohistochemical staining of MMP2 in the synovium of the right knee joint of mice and its mean optical density analysis, N = 6

To further determine the effect of DHJST on the cartilage tissue of KOA mice, the degradation of knee cartilage was evaluated by MMP-2 immunohistochemical staining and western blot detection of collagen 2 and collagen 4. The immunohistochemical staining results showed that compared with the normal group, the MMP-2 level in the cartilage tissue of the model group was significantly increased (P < 0.001). Treatment with DHJST (2 g/kg or 4 g/kg) significantly decreased MMP-2 in the cartilage tissues of model mice after 4 weeks (P = 0.0044, P < 0.0001) (Fig. 3A, B). Western blot results showed that treatment with DHJST (2 g/kg or 4 g/kg) increased the protein expression levels of collagen 2 and collagen 4 in the cartilage of KOA model mice (P < 0.0001) (Fig. 3C, D). In addition, immunohistochemical staining of synovial MMP2 showed that MMP-2 expression in the synovial tissue of mice in the model group was significantly increased (P < 0.001), and DHJST (2 g/kg or 4 g/kg) significantly decreased the expression level of MMP2 (P = 0.0070, P < 0.0001) in synovial tissues of mice after 4 weeks of treatment (Fig. 3E, F).

### DHJST inhibits Notch1 signaling and NLRP3 activation in the synovial tissue of KOA mice

**Fig. 4:**
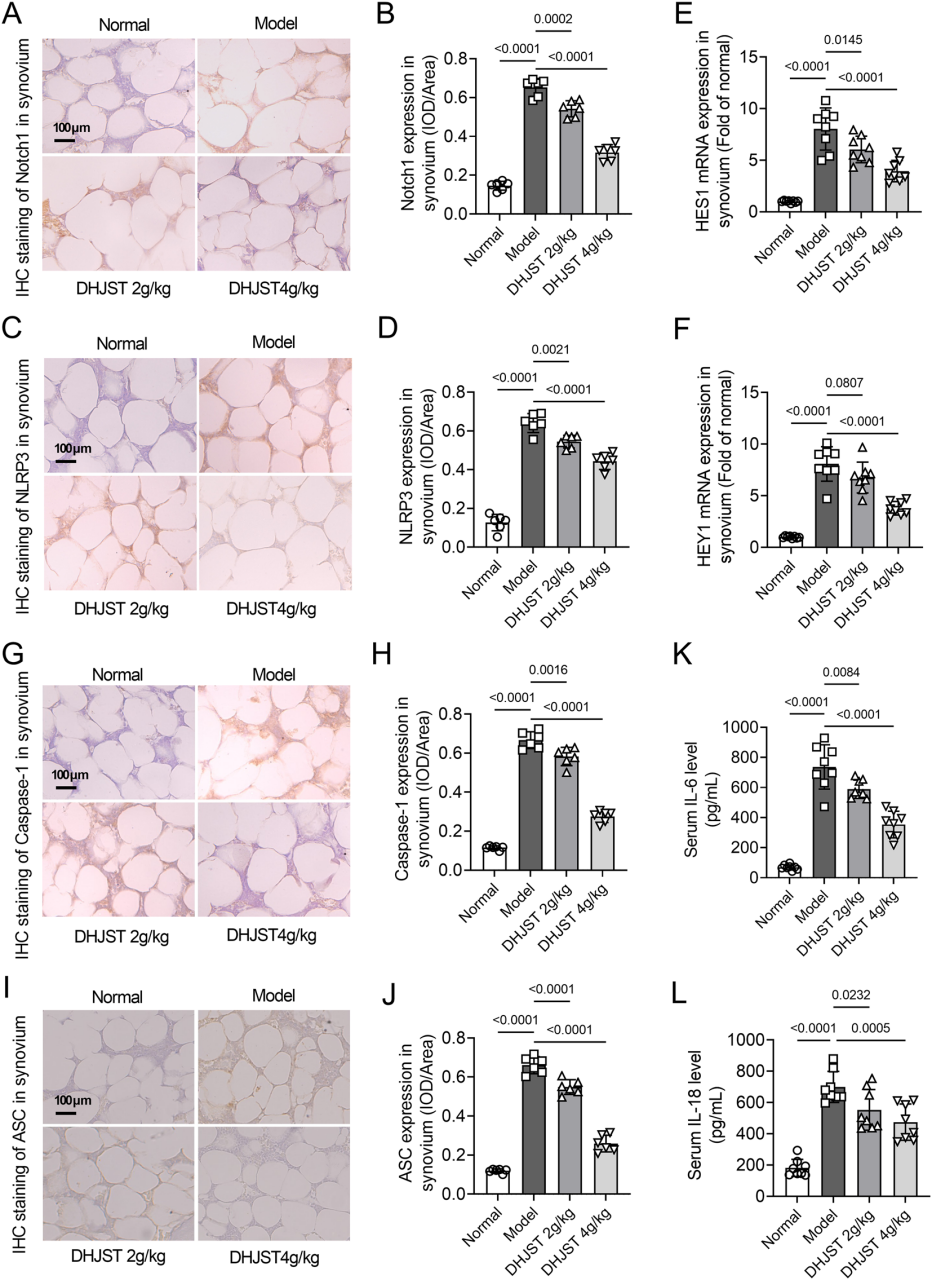
DHJST inhibits Notch1 signaling and NLRP3 activation in the synovial tissue of KOA mice C57BL/6 mice were injected with 4% papain 0.1 ml (once a week for three weeks) into the right knee joint to induce KOA. After that, they were treated with 2 g/kg or 4 g/kg DHJST for 4 weeks. **A**, **B** Immunohistochemical staining of Notch1 in the synovium of the right knee joint of mice and its mean optical density analysis. **C**, **D** Immunohistochemical staining of NLRP3 in the synovium of the right knee joint of mice and its mean optical density analysis. **E**, **F** mRNA expression levels of HES1 and HEY1 in the synovium of the right knee joint of mice. **G**, **H** Immunohistochemical staining of Caspase-1 in the synovium of the right knee joint of mice and its mean optical density analysis. **I**, **J** Immunohistochemical staining of ASC in the synovium of the right knee joint of mice and its mean optical density analysis. **K**, **L** Detection of serum IL-6 and IL-18 levels in mice by ELISA kit. N = 6 in **A**–**D** and **G**–**J**, N = 8 in **E**, **F** and **K**, **L**

To clarify the mechanism by which DHJST reduces cartilage damage in KOA mice, the expression levels of Notch1 and NLRP3 signaling in knee joint synovium were detected by immunohistochemical staining and western blotting. The immunohistochemical results showed that the expression levels of Notch1 and NLRP3 in the synovium of the model group were significantly increased compared with those of the normal group (P < 0.0001). After 4 weeks of treatment with DHJST (2 g/kg or 4 g/kg), the expression levels of Notch1 (P = 0.0002, P < 0.0001) and NLRP3 (P = 0.0021, P < 0.0001) in the synovium were significantly reduced (Fig. 4A–D). The results of real-time qPCR showed that the mRNA expression levels of HES1 and HEY1 in the synovium of the model group were significantly increased compared with those of the normal group (P < 0.001), while the mRNA expression levels of HES1 (P = 0.0145, P < 0.0001) and HEY1 (P = 0.0807, P < 0.0001) in the synovium of mice were significantly decreased after 4 weeks of treatment with DHJST (2 g/kg or 4 g/kg) (Fig. 4E, F). Moreover, the expressions of Caspase-1 and ASC in the synovium of the model group were significantly increased compared with those of the normal group (P < 0.0001). After 4 weeks of treatment with DHJST (2 g/kg or 4 g/kg), the expression levels of Caspase-1 (P = 0.0016, P < 0.0001) and ASC (< 0.0001, P < 0.0001) in the synovium were significantly decreased (Fig. 4G, J). Compared with the normal group, the serum IL-6 and IL-18 level of the mice in the model group was significantly increased, and the serum IL-6 and IL-18 level was significantly decreased after 4 weeks of treatment with DHJST (2 g/kg or 4 g/kg) (Fig. 4K-L).

### NLRP3 interference alleviates symptoms and increases the therapeutic effect of DHJST in KOA mice

**Fig. 5:**
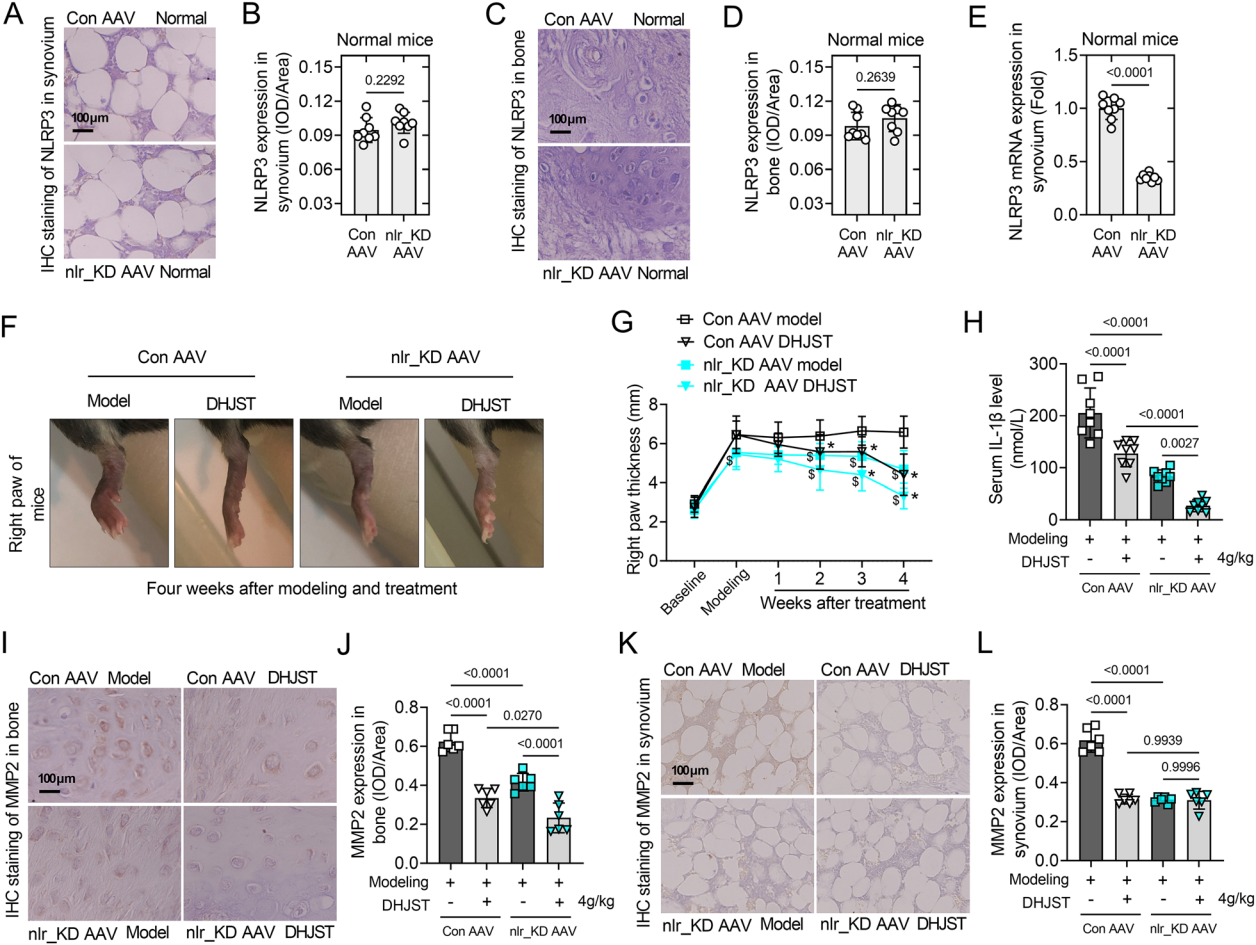
NLRP3 silencing alleviates symptoms and increases the therapeutic effect of DHJST in KOA mice C57BL/6 mice were injected with control AAV2 (Con AAV) or NLRP3 shRNA AAV2 (nlr_KD AAV) into the tail vein for 3 weeks. Then, 4% papain 0.1 ml (once a week for three weeks) was injected into the right knee joint of the mice to induce KOA, and 4 g/kg DHJST was used to treat the mice for 4 weeks. **A**, **B** Immunohistochemical staining of NLRP3 in mouse right knee joint synovium and mean optical density analysis in normal mice with Con AAV or nlr_KD AAV. **C**, **D** Immunohistochemical staining of NLRP3 in mouse right knee joint cartilage and its mean optical density analysis in normal mice with Con AAV or nlr_KD AAV. **E** NLRP3 mRNA expression levels in the synovium of the right knee joint of normal mice with Con AAV or nlr_KD AAV. **F** Representative picture of the morphology of the right knee joint of the mice after treatment. **G** Line plots recorded the thickness of the right paw of the mice before modeling (baseline), after modeling (modeling), and after weekly administration (1–4 weeks). Con AAV groups vs. nlr_KD AAV groups, $P < 0.05; DHJST groups vs. Model groups, * P < 0.05. (H) Detection of serum IL-1β levels in the mice after treatment by ELISA kit. **I**, **J** Immunohistochemical staining of MMP2 in mouse right knee joint cartilage and mean optical density analysis after treatment. **K**, **L** Immunohistochemical staining of MMP2 in mouse right knee joint synovium and its mean optical density analysis after treatment. N = 8 in **A**–**H**, N = 6 in **I**–**L**

To further verify the effect of NLRP3 on cartilage damage in KOA mice and to explore whether NLRP3 is involved in the therapeutic effect of DHJST on KOA mice. NLRP3 shRNA AAV was used to construct NLRP3 low expression (nlr_KD AAV) mice. Immunohistochemical staining showed that NLRP3 expression was quite low in the knee cartilage and synovium of normal mice with Con AAV infection, and almost no NLRP3 expression was found in the knee cartilage and synovium of normal mice with nlr_KD AAV infection (Fig. 5A–D). RT-PCR results showed that, compared with Con AAV infected normal mice, the mRNA expression of NLRP3 (P < 0.0001) were significantly decreased in normal mice with nlr_KD AAV infection (P < 0.001, Fig. 5E). Compared with the Con AAV model group, the right foot thickness of mice in the nlr_KD AAV model group was significantly reduced. In both Con AAV model mice and nlr_KD AAV model mice, DHJST showed a significant inhibitory effect on toe swelling in mice (P < 0.05, Fig. 5F, G). The expression levels of serum IL-1β (P < 0.0001), cartilage MMP2 (P < 0.0001) and synovial MMP2 (P < 0.0001) were significantly lower in mice in the nlr_KD AAV Model group than in those in the Con AAV model group (Fig. 5H–L). This suggests that low expression of NLRP3 directly alleviates the symptoms in KOA mice. In addition, in nlr_KD AAV model mice, DHJST further significantly reduced the serum IL-1β level (P = 0.0027) and the expression level of MMP2 in cartilage (P < 0.0001), while DHJST did not further reduce MMP2 in synovial membranes, probably due to the lower expression of MMP2 in nlr_KD AAV model mice (Fig. 5H–L). These results suggested that DHJST significantly inhibits the activation of NLRP3 in cartilage and circulating inflammation.

### NLRP3 interference did not affect the expression level of notch1 or downstream transcriptional signals in the synovia of KOA mice

**Fig. 6:**
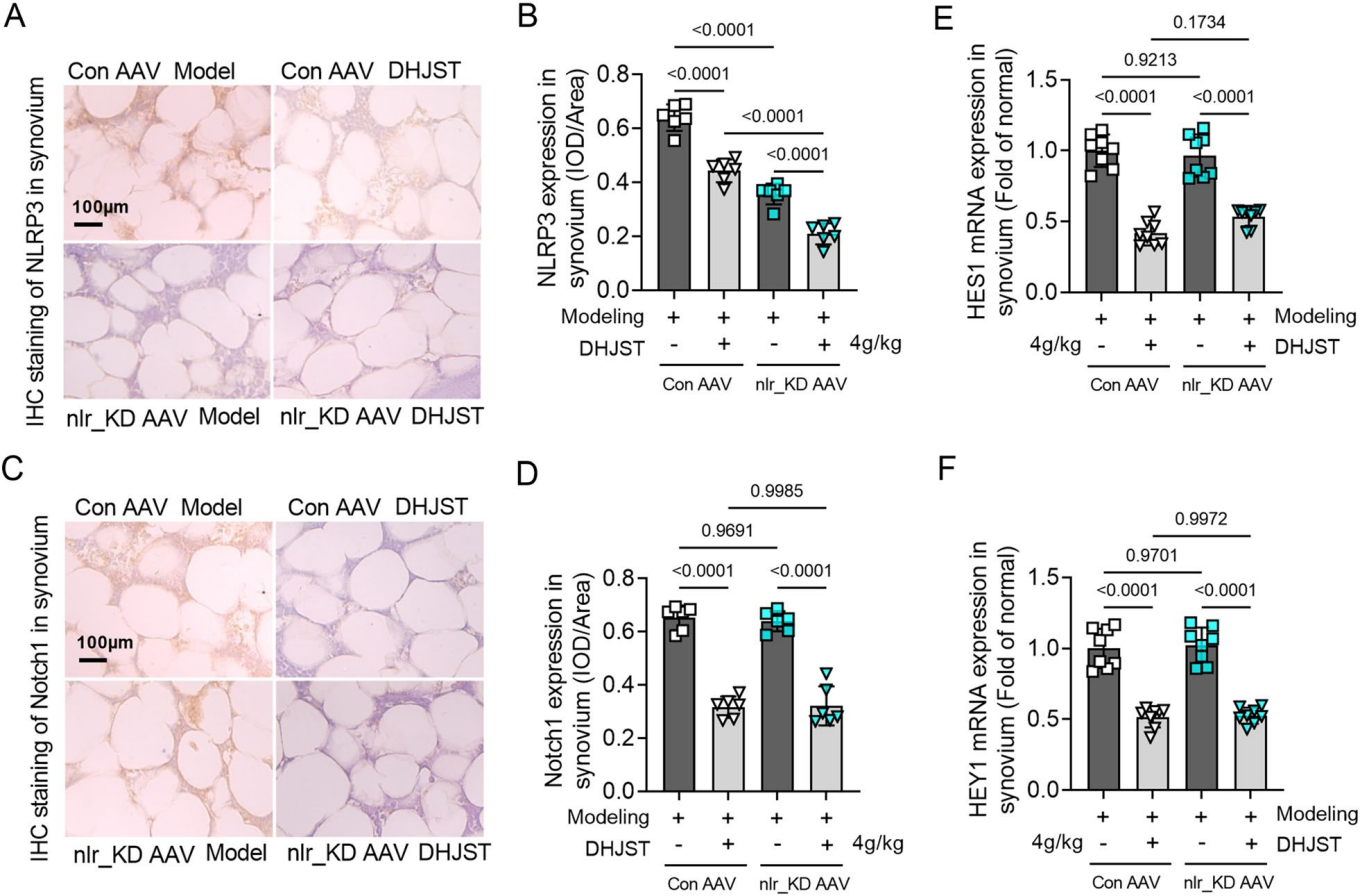
NLRP3 interference did not affect the expression level of notch1 or downstream transcriptional signals in the synovia of KOA mice C57BL/6 mice were injected with control AAV2 (Con AAV) or NLRP3 shRNA AAV2 (nlr_KD AAV) into the tail vein for 3 weeks. Then, 4% papain 0.1 ml (once a week for three weeks) was injected into the right knee joint of the mice to induce KOA, and 4 g/kg DHJST was used to treat the mice for 4 weeks. **A**, **B** Immunohistochemical staining of Notch1 in the synovium of the right knee joint of the mouse and its mean optical density analysis **C**, **D** Immunohistochemical staining of NLRP3 in the synovium of the right knee joint of the mouse and its mean optical density analysis. (E-F) mRNA expression levels of HES1 and HEY1 in the synovium of the right knee joint of mice. N = 6 in **A**–**D**, N = 8 in **E**–**F**

Immunohistochemical staining showed that the expression level of NLRP3 in the synovium of nlr_KD AAV mice was significantly lower than that of Con AAV mice, both in the knee joints of model mice (P < 0.0001) and DHJST-treated mice (P < 0.0001). In nlr_KD AAV model mice, DHJST further significantly reduced the expression level of NLRP3 in the synovium (P < 0.0001). nlr_KD AAV tail vein injection reduced the expression of systemic NLRP3 in mice, and DHJST significantly inhibited the activation of NLRP3 in the synovium (Fig. 6A-B). Compared with Con AAV mice, the expression level of Notch1 in the synovium of nlr_KD AAV mice did not change significantly, either in the model mice (P = 0.9691) or DHJST-treated mice (P = 0.9985). DHJST significantly reduced the level of Notch1 in the synovium of Con AAV model mice (P < 0.0001) and nlr_KD AAV model mice (P < 0.0001) (Fig. 6C-D). Similar changes also included changes in the mRNA expression levels of HES1 and HEY1 in the synovium (Fig. 6E, F). This suggests that the tail vein injection of nlr_KD AAV did not affect the expression level of notch1 in the joints of mice, and the interference of NLRP3 did not affect the inhibitory effect of DHJST on notch1 signaling in the synovium of mice.

### Notch1 overexpression abolishes the inhibitory effect of DHJST on cartilage degradation in KOA mice

**Fig. 7:**
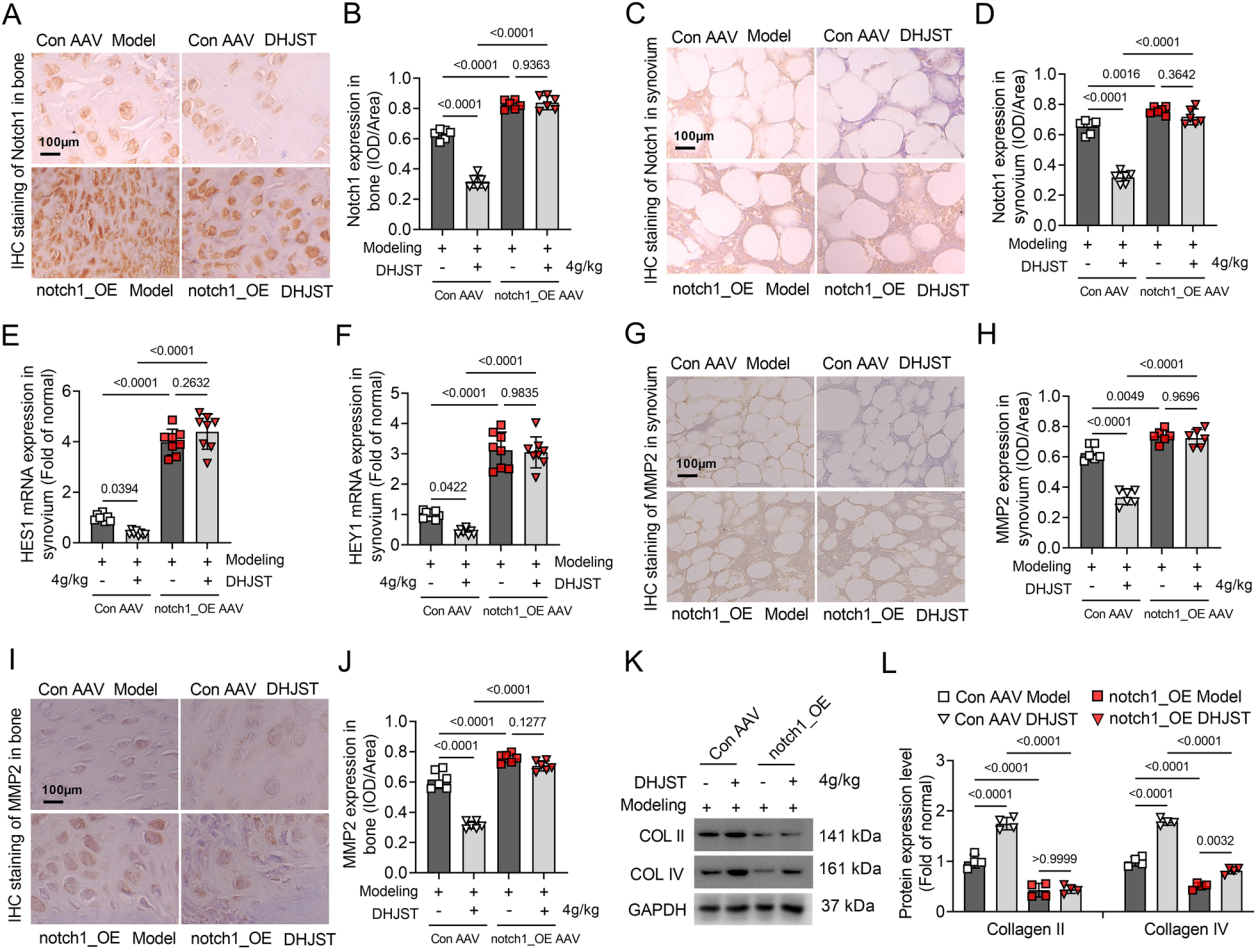
Notch1 overexpression abolishes the inhibitory effect of DHJST on cartilage degradation in KOA mice C57BL/6 mice were injected with control AAV2 (Con AAV) or Notch1-overexpressing AAV2 (notch1_OE AAV) into the tail vein for 3 weeks. Then, 4% papain 0.1 ml (once a week for three weeks) was injected into the right knee joint of the mice to induce KOA, and the mice were treated with 4 g/kg DHJST for 4 weeks. **A**, **B** Immunohistochemical staining of Notch1 in the right knee joint cartilage of the mice and its mean optical density analysis, N = 6. **C, D** Immunohistochemical staining of Notch1 in the synovial membrane of the right knee joint of the mice and its mean optical density analysis, N = 6. **E**, **F** mRNA expression levels of HES1 and HEY1 in the synovium of the right knee joint of mice, N = 8. **G**, **H** Immunohistochemical staining of MMP2 in the synovium of the right knee joint of mice and its mean optical density analysis, N = 6. **I**, **J** Immunohistochemical staining of MMP2 in the cartilage of the right knee joint of mice and mean optical density analysis, N = 6. **K, L** Western blot detection of collagen 2 and collagen 4 in the cartilage of the right knee joint of mice and their relative expression levels, N = 4

To further verify whether DHJST exerts a protective effect on cartilage damage in KOA mice by inhibiting Notch1. Notch1 overexpression (Notch1_OE AAV) mice were constructed. Immunohistochemical staining results showed that the expression level of Notch1 in the cartilage and synovium of Notch1_OE mice was significantly increased compared with that in Con AAV mice, both in the knee joint of model mice (P < 0.0001) and in DHJST-treated mice (P < 0.0001) (Fig. 7A–D). In Notch1_OE model mice, the inhibitory effect of DHJST on notch1 expression in cartilage (P = 0.9363) or synovium (P = 0.3642) was abolished (Fig. 7A–D). Similar changes in the mRNA expression levels of HES1 and HEY1 were observed in synovial membranes (Fig. 7E, F). These results indicate that the tail vein injection of Notch1_OE AAV increased the expression of Notch1 in the joints of mice and cancelled the inhibitory effect of DHJST on ntch1 signaling in KOA mice. On the other hand, compared with the Con AAV model group, the expression levels of cartilage MMP2 (P < 0.0001) and synovial MMP2 (P < 0.0001) in the Notch1_OE model group were significantly increased (Fig. 7G–J), while the protein expression levels of collagen 2 (P < 0.0001) and collagen 4 (P < 0.0001) in the knee cartilage tissue were significantly reduced (Fig. 7K, L). This suggests that the high expression of Notch1 promotes the symptoms of articular soft degradation in KOA mice. More importantly, in Notch1_OE KOA mice, the inhibitory effect of DHJST on the expression of synovial MMP2 (P = 0.9696) and cartilage MMP2 (P = 0.1277) and the upregulation of collagen 2 (P > 0.9999) in cartilage were abolished (Fig. 7G–L). Although DHJST can significantly upregulate the protein expression level of collagen 4 in knee cartilage in Notch1-overexpressing KOA mice (Fig. 7K, L), these results still suggest that DHJST protects articular cartilage in KOA mice by inhibiting the Ntoch1 signaling pathway.

### Notch1 overexpression abolishes the inhibitory effect of DHJST on synovial NLRP3 activation in KOA mice

**Fig. 8:**
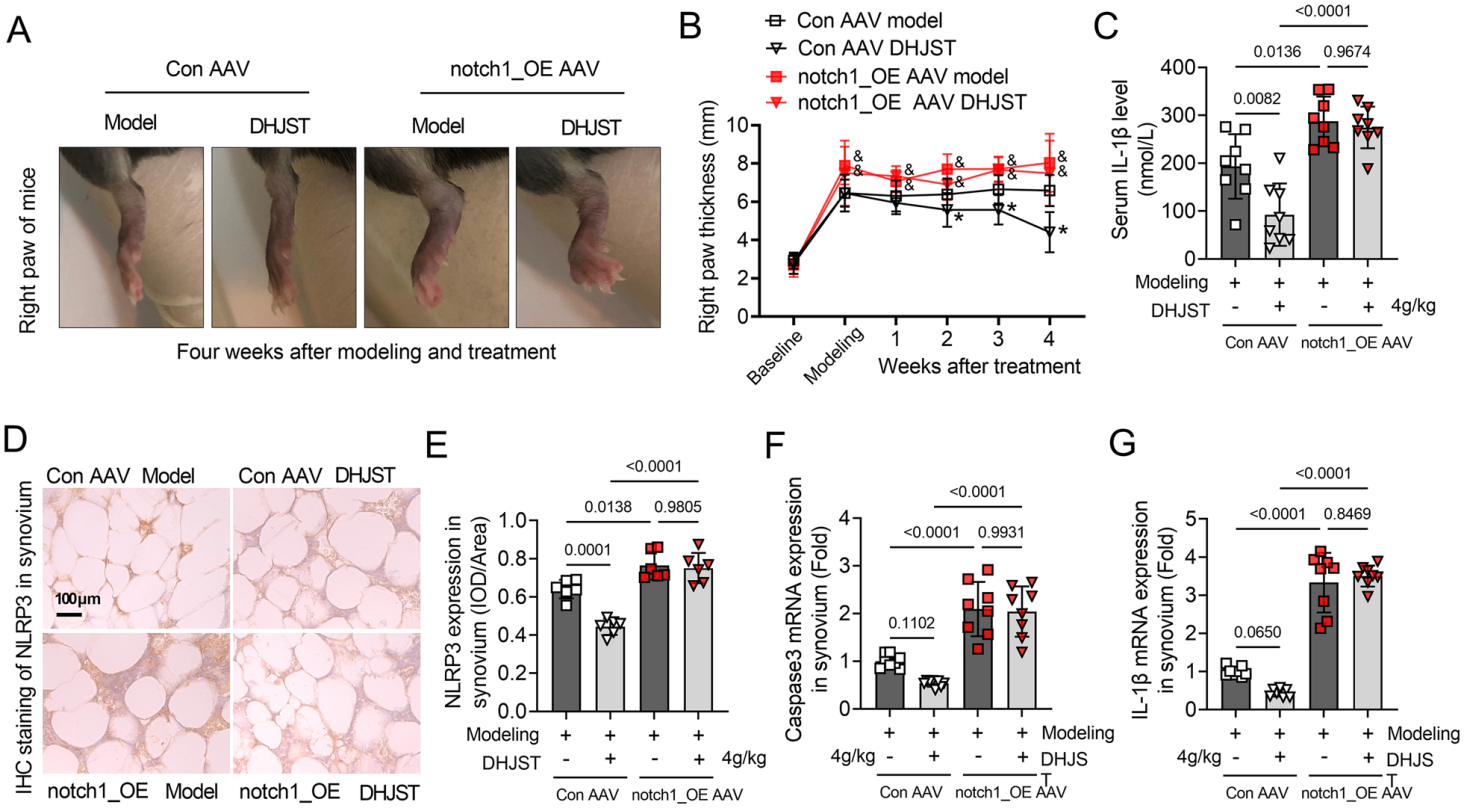
Notch1 overexpression abolishes the inhibitory effect of DHJST on synovial NLRP3 activation in KOA mice C57BL/6 mice were injected with control AAV2 (Con AAV) or Notch1-overexpressing AAV2 (notch1_OE AAV) into the tail vein for 3 weeks. Then, 4% papain 0.1 ml (once a week for 3 weeks) was injected into the right knee joint of the mice to induce KOA, and 4 g/kg DHJST was used to treat the mice for four weeks. **A** A representative picture of the morphology of the right knee joint of the mice after treatment. **B** Line plots recorded the thickness of the right paw of the mice before modeling (baseline), after modeling (modeling), and after weekly administration (1–4 weeks). Con AAV groups vs. notch1_OE AAV groups, & P < 0.05; DHJST groups vs. Model groups, * P < 0.05. **C** Serum IL-1β levels in mice were detected by ELISA. (D-E) Immunohistochemical staining of NLRP3 in the synovium of the right knee joint of mice and mean optical density analysis. **F, G** mRNA expression levels of Caspase3 and IL-1β in the synovium of the right knee joint of mice. N = 6 in **D**, **E**; N = 8 in **A**–**C** and **F**, **G**.

The right paw thickness was significantly increased in the Notch1_OE model group of mice compared with the Con AAV model group (P < 0.05). In contrast, DHJST showed no inhibitory effect on the paw thickness of KOA mice with Notch1_OE, and the mice still showed significant swelling of the toes compared with the Con AAV model group (Fig. 8A, B). Serum IL-1β levels were significantly increased in the Notch1_OE model group compared to the Con AAV model group (P = 0.0136), while DHJST failed to reduce IL-1β levels in the KOA serum of Notch1_OE KOA mice (P = 0.9674) (Fig. 8C). These results suggest that tail vein injection of Notch1_OE AAV promoted joint swelling and inflammation in KOA mice and abolished the alleviating effect of DHJST on related symptoms in KOA mice. On the other hand, compared with the Con AAV Model group, the expression level of NLRP3 (P = 0.0138) in the synovial membrane of the Notch1_OE model group was significantly increased (Fig. 8D-E), and the mRNA expression levels of Caspase3 (P < 0.0001) and IL-1β (P < 0.0001) in the synovial tissue of the knee joint were also significantly increased (Fig. 8F, G). This suggests that the high expression of Notch1 promotes the activation of NLRP3 signaling in the knee joint of KOA mice. More importantly, in Notch1_OE KOA mice, DHJST inhibited the expression of synovial NLRP3 (P = 0.9805) protein, and the expression of Caspase3 (P = 0.9931) and IL-1β (P = 0.8469) mRNA was abolished (Fig. 8D–G). These results indicate that DHJST has a therapeutic effect on KOA mice by inhibiting Ntoch1 signaling and its subsequent NLRP3 signaling activation.

## Discussion

As a common joint disease, the incidence of KOA has increased in recent years [16]. Previous studies have shown that various forms of inflammation and cartilage damage promote the progression of KOA [17]. Our recent study showed that DHJST can alleviate cartilage damage in a rat KOA model by inhibiting NLRP3 and reduce serum inflammation in KOA patients [8]. However, it is unclear how DHJST inhibits cartilage damage or mitigates the progression of KOA by inhibiting NLRP3. In the current study, we first confirmed that DHJST improved toe swelling, knee cartilage damage, and inflammation in a KOA mouse model. Furthermore, we observed that DHJST reduced the level of MMP2 in knee cartilage and synovial rental in KOA mice, suggesting that DHJST reduced cartilage degradation.

Cartilage degeneration is the most common pathological change in KOA, where chondrocytes (the only cell type in cartilage) are synthesized and encapsulated in the extracellular matrix [18, 19]. Inflammation usually stimulates their activation and reduces the number of chondrocytes, which directly leads to articular cartilage damage [20]. Synovial inflammation is one of the important causes of cartilage degeneration. IL-1β involved in cartilage degradation may be produced by synovial cells rather than chondrocytes. The protein level of NLRP3 in the synovium of KOA patients is more than 5.4 times higher than that of normal patients [21]. Synovial uric acid levels were found to be closely related to synovial IL-18 and IL-1β in the synovial fluid of patients with KOA, and it is believed that synovial uric acid can be used as one of the markers to evaluate the severity of KOA [22]. In addition, genetic studies have demonstrated that genes related to the NLRP3 inflammatory signaling pathway (including NLRP3, ASC, and Caspase-1) are significantly upregulated in RA synovial tissues [23]. We confirmed the high expression status of NLRP3 in synovial tissues of KOA mice after assaying the expression level of NLRP3 in synovial membranes and confirmed the inhibitory effect of DHJST on synovial NLPR3.

DHJST has been used for treating arthritis in China for over 1300 years, but preclinical studies on its therapeutic effects in KOA are limited. Glycyrrhetinic acid, citric acid, paeoniflorin, and gentioline are all monomeric substances in DHJST and are commonly used as quality control standards for DHJST. Glycyrrhizic acid has been reported to suppress inflammation in chondrocytes [24], while paeoniflorin has been reported to inhibit MMP secretion and NF-κB activation in chondrocytes induced by IL-1β [25]. Choline has been reported to have therapeutic effects on arthritis in rats [26]. In this study, we first demonstrated the significant therapeutic effects of DHJST on KOA mice, especially by inhibiting NLRP3 to reduce the secretion of MMP and inflammatory factors in the cartilage and synovial membrane.

Furthermore, after constructing NLRP3 low expression mice, we found a significant reduction in knee swelling and MMP2 expression in articular cartilage and synovial membranes caused by papain knee injection, which confirmed that the NLRP3 inflammasome is a promising target for KOA treatment. Studies have shown that different modalities of inhibition of NLRP3 activation alleviate KOA progression [27–30]. These results at least partially suggested that DHJST exerts a therapeutic effect on KOA by inhibiting NLRP3. On the other hand, we found that in nlr_KD AAV model mice, DHJST further reduced the serum IL-1β level and the expression level of MMP2 in cartilage, indicating that DHJST can further inhibit the expression of NLRP3 in nlr_KD AAV model mice, which means DHJST can reduce inflammation through other pathways as well. DHJST is a complex mixture of multiple herbs, and previous studies have shown that many of the individual herbs in DHJST have anti-KOA effects through various mechanisms, such as inhibiting NF-κB and MAPK signaling pathways, suppressing cytokine production, and reducing oxidative stress [31]. Further investigation is required to elucidate the underlying mechanism of DHJST in NLRP3 and other pathways.

The involvement of the NLRP3 inflammasome in KOA cartilage degeneration, synovial inflammation, and pain has been widely recognized [32, 33]. It is necessary to further clarify the mechanism of NLRP3 activation and regulation in KOA and confirm the target of DHJST. Recent studies have identified Notch1 as a novel activator of NLRP3 inflammasome signaling, leading to chronic tissue injury and myofibroblast differentiation in keloid progression [34]. We found that not only the expression of NLRP3 but also the expression of Notch1 in the synovial tissue of KOA mice was significantly increased. Importantly, nlr_KD AAV decreased the expression of NLRP3 in KOA mice, but the expression levels of Notch1 in the synovial membranes of nlr_KD AAV KOA mice, including the mRNA expression levels of the transcription factors HES1 and HEY1, which are activated by Notch1, were not significantly changed. This suggests that Notch1 may be an upstream factor regulating NLRP3 in KOA model mice. On the other hand, DHJST had the same effect on notch1 signaling in the synovium of mice with or without NLRP3 interference, which not only suggested that Notch1 might be an upstream factor of NLRP3 in KOA model mice but also suggested that DHJST might play a regulatory role in NLRP3 by inhibiting notch1 signaling.

To further prove the mechanism of the protective effect of DHJST on KOA in vivo, we constructed Notch1-overexpressing mice and established a C57BL/6 mouse model of KOA in their bodies by papain knee injection. As expected, Notch1 overexpression promoted cartilage degradation in KOA mice. The expression level of MMP2 in both the synovium and cartilage tissue was significantly increased compared with that in control AAV-infected KOA mice. More importantly, DHJST lost its therapeutic effect on Notch1-overexpressing KOA mice. Its inhibitory effect on MMP2 expression in joints and cartilage, reduction of toe swelling, and reduction of serum IL-1β in KOA model mice were all abolished by Nothc1 overexpression. On the other hand, the activation of NLRP3 in the synovial membrane of KOA mice was increased after Notch1 overexpression, suggesting that Notch1 overexpression promotes inflammation by upregulating NLRP3 expression during the development of KOA. Notably, the inhibitory effect of DHJST on Notch1 and NLRP3 expression was also abolished in Notch1-overexpressing KOA mice. These results indicated that DHJST plays a regulatory role in NLRP3 by inhibiting notch1 signaling in KOA mice.

## Conclusion

Our findings confirm the protective effect of DHJST on KOA in mice. In addition, we found that DHJST exerts its NLRP3 inhibitory and anti-inflammatory effects by inhibiting Notch1 signaling in the joint. These findings illustrate that DHJST is a promising drug for the treatment of KOA and reveal the potential mechanism underlying its KOA effects.

## Data Availability

The data are available from the corresponding author on reasonable request.

## Abbreviations

DHJST: Du Huo Ji Sheng Tang
IL-1β: interleukin-1β
KOA: Knee osteoarthritis
MMP: Matrix metalloproteinase
OA: Osteoarthritis
EDTA: Ethylenediaminetetraacetic acid
H&E: Hematoxylin and Eosin
NLRP3: NLR family pyrin domain containing 3
Caspase-1: cysteinyl aspartate specific proteinase-1
ASC: apoptosis-associated speck-like protein containing a CARD
NF-κB: nuclear factor kappa-B.
